# TG/HDL-C ratio is positively associated with risk and severity of CHD among NAFLD patients: a case control study

**DOI:** 10.3389/fendo.2024.1383489

**Published:** 2024-07-04

**Authors:** Biwei Cheng, Yumeng Yi, Mingtai Chen, Yi Wei, Xuekang Su, Peiying Chen, Xiaojuan Lin, Yanghui Gu, Tao Li, Chong Xu, Qiang Liu, Biao Li

**Affiliations:** ^1^ Shenzhen Traditional Chinese Medicine Hospital, Shenzhen, Guangdong, China; ^2^ The Fourth Clinical Medical College of Guangzhou University of Chinese Medicine, Guangzhou, China

**Keywords:** triglyceride, high-density lipoprotein cholesterol, insulin resistance, nonalcoholic fatty liver disease, coronary heart disease

## Abstract

**Objective:**

This study aimed to explore the association between the triglyceride to high-density lipoprotein cholesterol (TG/HDL-C) ratio and the risk and severity of CHD among NAFLD patients.

**Methods:**

This retrospective study included 278 patients with NAFLD and chest pain. The TG/HDL-C ratio was calculated and coronary angiography performed. All individuals were divided into NAFLD + CHD and NAFLD groups. The severity of coronary artery stenosis is quantified using the Gensini score based on angiographic results. In NAFLD patients, the association between the TG/HDL-C ratio and the risk and severity of CHD was explored.

**Results:**

CHD was detected in 139 of 278 patients. Compared to NAFLD group, multivariate logistic regression showed that TG/HDL-C ratio was a risk factor for CHD among NAFLD patients after adjustment for confounding factors with the odds ratio (OR 1.791, 95% CI 1.344–2.386, P<0.001). Further analysis using multivariate logistic regression based on tertiles revealed that, after adjusting for confounding factors, compared to the T1 group, the risk of CHD in the T2 group was 2.17-fold higher (OR, 2.17; 95% CI, 1.07–4.38; P = 0.031). Similarly, the risk of CHD in the T3 group increased by 2.84-fold (OR, 2.84; 95% CI, 1.36–5.94; P = 0.005). The multifactor linear regression analysis showed each 1-unit increase in TG/HDL-C ratio in the NAFLD + CHD group was associated with a 7.75-point increase in Gensini score (β=7.75, 95% CI 5.35–10.15, P<0.001).

**Conclusion:**

The TG/HDL-C ratio was positively correlated with CHD risk and reflected coronary atherosclerosis severity in NAFLD patients.

## 1 Introduction

Coronary heart disease (CHD) is one of the leading causes of death worldwide (1, 2). Hypertension (HTN), diabetes, hyperlipidemia, obesity, and smoking are well-recognized risk factors for CHD. However, studies have found that CHD can still progress in some patients, even when the known risk factors are controlled well. This underscores the critical importance of identifying and managing the residual risks of CHD (3).

Non-alcoholic fatty liver disease (NAFLD), a metabolic disorder affecting the liver, is associated to an elevated risk of cardiovascular disease, although its precise mechanism remains unclear (4).

Insulin resistance (IR) contributes to atherosclerotic plaque formation and elevates the risk of CHD (5). Furthermore, reports indicate a robust association between IR and the progression of NAFLD. However, the correlation between IR and the risk as well as severity of CHD among NAFLD patients is still unclear.

While the high insulin-glucose clamp test is currently the gold standard for diagnosing insulin resistance, its clinical application is often restricted. Alternatively, the triglyceride to high-density lipoprotein cholesterol (TG/HDL-C) ratio has been deemed as a dependable indicator of IR (6). Studies have reported an association between the TG/HDL-C ratio and both increased risk and severity of coronary heart disease (7, 8).

This study aims to compare TG/HDL-C ratios among patients with NAFLD. Furthermore, it investigates the association between insulin resistance and the risk and severity of CHD.

## 2 Methods

### 2.1 Subjects and study design

A total of 278 patients with NAFLD were included in this retrospective study. All patients underwent coronary angiography for chest pain between April 2021 and August 2023 at Shenzhen Traditional Chinese Medicine Hospital. All participants were categorized into two groups: NAFLD + CHD and NAFLD, based on the results of coronary angiography.

The diagnostic criteria for NAFLD are outlined in the 2018 Liver Disease Clinic Guidelines (9). Specifically, patients must have no history of alcohol consumption or consume less than 140 g/week of equivalent ethanol (less than 70 g/week for females). Abdominal ultrasound is utilized for assessment, wherein the presence of two out of the following three manifestations indicates diffuse fatty liver disease: (a) Diffuse enhancement of near-field echogenicity in the liver, with echogenicity stronger than that of the renal cortex; (b) Poor visualization of intrahepatic ductal structures; (c) Gradual attenuation of far-field echogenicity in the liver.

The Judkin method (10) was used to record the coronary angiography results, and coronary heart disease was diagnosed when the lumen diameter of the main coronary artery or other vital branches showed stenosis of ≥50%. Gensini Score (GS) is a widely used angiographic scoring system for quantifying the severity of CAD. The GS has been developed to characterize the complexity of CAD taking into consideration 3 main parameters for each coronary lesion: severity score, region multiplying factor and collateral adjustment factor (11, 12). Therefore, in this study, the severity of coronary arteries was quantified using the Gensini Score. Two independent cardiologists, blinded to the study protocol and patient details, determined the Gensini Score via preoperative angiography.

### 2.2 Exclusion criteria

1. Patients with cerebrovascular disease, aortic disease, or who have had a cerebral infarction within the last six months;
2. Patients with severe liver dysfunction, those who have undergone unilateral nephrectomy, or with a malignant tumor or autoimmune disease;
3. Patients who have undergone surgery or a bypass procedure within the previous 3 months;
4. Patients with a urinary tract infection or lung infection within the past month;
5. Pregnant and lactating women, female patients who intend to become pregnant;
6. Patients with mental illness or limited mobility who are unable to cooperate with the examination;
7. Patients with previous stent implantation;
8. Specific diseases known to cause fatty liver, such as viral, drug-induced, genetic, and autoimmune liver diseases.
9. Incomplete clinical data collection.

## 3 Data collection and measurements

General clinical information, including age, sex, hypertension (HTN) history, diabetes mellitus (DM) history, smoking history, systolic blood pressure (SBP), diastolic blood pressure (DBP), heart rate (HR), and body mass index (BMI), was collected via the electronic medical record system.

Biochemical parameters such as alanine aminotransferase (ALT), aspartate aminotransferase (AST), serum creatinine (Scr), total cholesterol (TC), triglycerides (TG), high-density lipoprotein cholesterol (HDL-C), low-density lipoprotein cholesterol (LDL-C), and fasting blood glucose (FBG) were obtained from the patients. The TG/HDL-C ratio was calculated. Based on the imaging results, acute myocardial infarction (AMI), acute coronary syndrome (ACS), and the number of stents were recorded, and the Gensini score was calculated.

## 4 Statistical analysis

All data were analyzed using SPSS Statistics version 27.0. And R 4.3 was also used for plotting the ROC. The Shapiro-Wilk test was used to assess the normality of continuous variable distributions. Normally distributed data were presented as mean ± standard deviation, whereas non-normally distributed data were expressed as median (P25, P75). Categorical variables were described in terms of frequency and percentage (%). The Student’s t test and one-way analysis of variance were used for continuous variables, and the chi-square test or Fisher’s exact probability method was used for categorical variables.

Initially, univariate logistic regression analysis was employed to explore the potential predictors of CHD. Subsequently, multivariate analysis was conducted to determine the independent predictors and their effect sizes. Baseline characteristics of tertiles of the TG/HDL-C ratio were compared using t-tests or one-way analysis of variance for continuous variables. For categorical variables, the chi-square test or Fisher’s exact test was applied. The predictive capacity of different TG/HDL-C ratio thresholds for CHD was assessed using receiver operating characteristic (ROC) curve analysis and the corresponding area under the curve (AUC) values.

## 5 Results

### 5.1 Baseline characteristics

Data were generated from 278 patients with NAFLD (155 men and 123 women), including 139 participants with CHD and 139 participants without CHD. The mean ages of the NAFLD+CHD and NAFLD groups were 59.89 ± 11.47 years and 57.15 ± 11.76 years, respectively. **Table 1** presents the baseline characteristics of both groups. There were no statistically significant differences between the two groups in terms of BMI, HTN, SBP, DBP, ALT, AST, TC, LDL-C, and HR (all P > 0.05). The proportion of males, smokers, and patients with DM was higher in the NAFLD+CHD group. Additionally, the NAFLD+CHD group had a higher mean age, Scr, TG, FBG, and TG/HDL-C ratio, as well as lower HDL-C levels, compared to the NAFLD group (all P < 0.05).

**Table 1:** Baseline characteristics of NAFLD+CHD group vs. NAFLD group.

	CHD + NAFLD (n = 139)	NAFLD (n = 139)	P
Gender (male)	91 (65.5%)	64 (46.0%)	0.001
Age (years)	59.89 ± 11.47	57.15 ± 11.76	0.050
BMI (kg/m2)	25.27 ± 2.94	25.04 ± 3.52	0.554
Smoke [ (n%)]	44 (31.7%)	25 (18.0%)	0.008
HTN [ (n%)]	95 (68.3%)	89 (64.0%)	0.447
DM [ (n%)]	52 (37.4%)	36 (25.9%)	0.039
SBP (mmHg)	138.77 ± 17.11	137.41 ± 22.93	0.576
DBP (mmHg)	86.19 ± 11.45	85.90 ± 15.38	0.860
ALT (U/L)	20.30 (14.10–32.30)	20.30 (14.30–28.30)	0.844
AST (U/L)	20.00 (15.90–25.20)	20.00 (15.30–31.20)	0.398
Scr (mmol/L)	82.57 ± 25.26	72.38 ± 18.81	<0.001
TC (mmol/L)	4.59 ± 1.11	4.42 ± 1.00	0.187
TG (mmol/L)	1.80 (1.26–2.82)	1.38 (1.00–2.00)	<0.001
HDL-C (mmol/L)	1.05 ± 0.26	1.17 ± 0.24	<0.001
LDL-C (mmol/L)	2.87 ± 0.95	2.81 ± 0.91	0.581
FGB (mmol/L)	6.14 ± 2.29	5.55 ± 1.59	0.013
HR (bpm)	80.80 ± 13.18	82.47 ± 14.70	0.318
TG/HDL-C	1.89 (1.17–3.08)	1.23 (0.79–1.99)	<0.001

Continuous variables with a normal distribution were reported as mean ± standard deviation (SD), whereas skewed continuous variables were presented as medians (P25-P75). Categorical data are presented as n (%). BMI, body mass index; HTN, hypertension; DM, diabetes mellitus; SBP, systolic blood pressure; DBP, diastolic blood pressure; ALT, alanine aminotransferase; AST, aspartate aminotransferase; Scr, serum creatinine; TC, total cholesterol; TG, triglycerides; HDL-C, high-density lipoprotein cholesterol; LDL-C, low-density lipoprotein cholesterol; FBG, fasting blood glucose; HR, heart rate; TG/HDL-C, the triglyceride to high-density lipoprotein cholesterol rate.The results demonstrated Gender, Smoke, DM, Scr, TG, HDL-C, FGB and TG/HDL-C rate are different between the two groups (P<0.05).

### 5.2 Univariate and multivariate logistic regression analysis predicting NAFLD with CHD

The univariate logistic regression analysis revealed that sex, smoking, DM, AST, Scr, TG, HDL-C, FBG, and the TG/HDL-C ratio were significantly associated with the occurrence of coronary heart disease in patients with NAFLD (all P < 0.05). In the subsequent multivariate logistic regression analysis, sex, age, DM, AST, Scr, FBG, and the TG/HDL-C ratio were included as predictors, while TG and HDL-C, which constitute the TG/HDL-C ratio, were excluded to prevent potential interactions. Multivariate logistic regression analysis demonstrated that age (OR = 1.042, 95% CI = 1.015–1.069, P = 0.002) and the TG/HDL-C ratio (OR = 1.791, 95% CI = 1.344–2.386, P < 0.001) were still risk factors for CHD in NAFLD patients (**Table 2**).

**Table 2:** Univariate and multivariate logistic regression analysis of coronary heart disease risk in patients with NAFLD.

	Univariate analysis			Multivariate analysis		
	OR	95% CI	P	OR	95% CI	P
Gender (male)	2.222	1.370–3.602	0.001	1.752	0.871–3.527	0.116
Age (years)	1.021	1.000–1.042	0.051	1.041	1.015–1.069	0.002
BMI (kg/m2)	1.022	0.950–1.100	0.552			
Smoke [(n%)]	2.112	1.205–3.702	0.009	1.479	0.731–2.993	0.277
HTN [(n%)]	1.213	0.737–1.995	0.447			
DM [(n%)]	1.710	1.025–2.853	0.040	0.978	0.479–1.997	0.951
SBP (mmHg)	1.003	0.992–1.015	0.574			
DBP (mmHg)	1.002	0.984–1.019	0.859			
ALT (U/L)	1.005	0.991–1.019	0.491			
AST (U/L)	1.019	1.003–1.035	0.017	1.011	0.996–1.026	0.148
Scr (mmol/L)	1.022	1.010–1.034	<0.001	1.011	0.997–1.025	0.132
TC (mmol/L)	1.163	0.929–1.455	0.187			
TG (mmol/L)	2.012	1.510–2.679	<0.001			
HDL-C (mmol/L)	0.128	0.046–0.358	<0.001			
LDL-C (mmol/L)	1.074	0.833–1.385	0.580			
FGB (mmol/L)	1.178	1.029–1.348	0.017	1.100	0.922–1.311	0.291
HR (bpm)	0.991	0.975–1.008	0.317			
TG/HDL-C	1.944	1.502–2.515	<0.001	1.789	1.357–2.359	<0.001

OR, odd ratio; CI, confidence interval; BMI, body mass index; HTN, hypertension; DM, diabetes mellitus; SBP, systolic blood pressure; DBP, diastolic blood pressure; ALT, alanine aminotransferase; AST, aspartate aminotransferase; Scr, serum creatinine; TC, total cholesterol; TG, triglycerides; HDL-C, high-density lipoprotein cholesterol; LDL-C, low-density lipoprotein cholesterol; FBG, fasting blood glucose; HR, heart rate; TG/HDL-C, the triglyceride to high-density lipoprotein cholesterol rate.

The univariate logistic regression analysis indicated TG/HDL-C ratio is a risk factors for CHD risk in NAFLD patients.

And the further multivariate logistic regression analysis indicated TG/HDL-C ratio is independent risk factors for CHD risk in NAFLD patients.

### 5.3 The predictive performance of the TG/HDL-C ratio for CHD in patients with NAFLD

**Figure 1:**
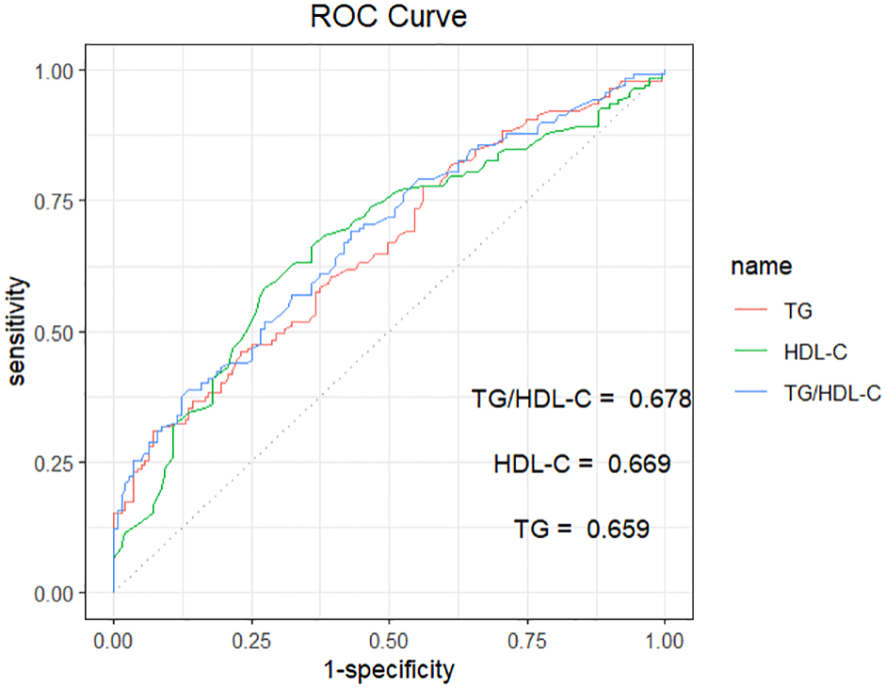
ROC curve analysis of TG、HDL-C、TG/HDL-C in predicting NAFLD with CHD. TG, triglycerides; HDL-C, high-density lipoprotein cholesterol; TG/HDL-C, the triglyceride to high-density lipoprotein cholesterol rate. The AUC for TG, HDL-C, and TG/HDL-C were 0.659, 0.669, and 0.678, respectively.

ROC curves were plotted using whether CHD occurred in NAFLD as the state variable. The results showed that the area under the curve (AUC) for TG was 0.659 (95% CI: 0.596-0.722, P<0.001), with a sensitivity of 0.309 and a specificity of 0.928. The AUC for HDL-C was 0.669 (95% CI: 0.605-0.733, P<0.001), with a sensitivity of 0.583 and specificity was 0.727. The AUC for the TG/HDL-C ratio was 0.677 (95% CI: 0.615-0.740, P<0.001), with a sensitivity of 0.381 and a specificity of 0.878 (**Table 3**; **Figure 1**).

**Table 3:** ROC curve analysis of TG, HDL-C, TG/HDL-C.

	AUC (95% CI)	P	Sensitivity	Specificity	Youden’s index	Cutoff
TG	0.659 (0.596–0.722)	<0.001	0.309	0.928	0.237	2.615
HDL-C	0.669 (0.605–0.733)	<0.001	0.583	0.727	0.309	1.025
TG/HDL-C	0.678 (0.615–0.740)	<0.001	0.381	0.878	0.259	2.422

ROC, the receiver operating characteristic; AUC, area under the curve; TG, triglycerides; HDL-C, high-density lipoprotein cholesterol; TG/HDL-C, the triglyceride to high-density lipoprotein cholesterol rate.

### 5.4 The baseline characteristics based on tertiles of the TG/HDL-C

Baseline information based on the tertiles of the TG/HDL-C ratio is shown in **Table 4**. Compared to the T1 group, a higher proportion of patients with elevated TG/HDL-C ratios were male. These patients exhibited increased prevalence rates of DM, CHD, ACS, and AMI. Furthermore, they underwent more stent implantations. They also had higher BMI, SBP, ALT, AST, Scr, TC, TG, FGB, cTnI levels, and Gensini scores. Additionally, they had lower HDL-C levels (all P < 0.05).

**Table 4:** Baseline Characteristics Stratified by Tertiles of the TG/HDL-C Ratio.

	T1 (n=93)	T2 (n=92)	T3 (n=93)	P
Gender (male)	40 (43.0%)	55 (59.8%)	60 (64.5%)	0.008
Age (years)	60.08 ± 10.63	58.45 ± 12.25	57.04 ± 12.01	0.208
BMI (kg/m2)	24.31 ± 3.20	25.53 ± 3.18	25.63 ± 3.19	0.008
Smoke [ (n%)]	16 (17.2%)	23 (25.0%)	30 (32.3%)	0.059
HTN [ (n%)]	55 (59.1%)	66 (71.7%)	63 (67.7%)	0.18
DM [ (n%)]	19 (20.4%)	31 (33.7%)	38 (40.9%)	0.010
SBP (mmHg)	133.78 ± 18.26	141.87 ± 20.50	138.66 ± 21.15	0.023
DBP (mmHg)	83.46 ± 11.40	86.71 ± 13.91	87.97 ± 14.81	0.064
ALT (U/L)	15.50 (12.00–22.65)	20.60 (14.93–29.98)	24.30 (17.10–39.95)	<0.001
AST (U/L)	17.30 (14.60–24.60)	20.85 (15.83–26.08)	20.90 (16.40–33.10)	<0.001
Scr (mmol/L)	70.18 ± 16.70	79.57 ± 25.39	82.70 ± 23.75	<0.001
TC (mmol/L)	4.30 ± 0.91	4.33 ± 0.99	4.87 ± 1.17	<0.001
TG (mmol/L)	1.00 (0.85–1.15)	1.60 (1.34–1.80)	2.75 (2.35–3.50)	<0.001
HDL-C (mmol/L)	1.33 ± 0.25	1.06 ± 0.17	0.94 ± 0.16	<0.001
LDL-C (mmol/L)	2.75 ± 0.87	2.80 ± 0.89	2.98 ± 1.02	0.233
FGB (mmol/L)	5.07 (4.70–5.49)	5.42 (4.73–6.53)	5.45 (4.97–6.67)	0.012
HR (bpm)	81.44 ± 13.95	81.07 ± 14.35	82.40 ± 13.69	0.800
cTnI, ng/ml	0.01 (0.01–0.01)	0.01 (0.01–0.01)	0.01 (0.01–0.15)	<0.001
CHD[ (n%)]	30 (32.3%)	48 (52.2%)	61 (65.6%)	<0.001
AMI[ (n%)]	3 (3.2%)	13 (14.1%)	23 (24.7%)	<0.001
ACS[ (n%)]	4 (4.3%)	17 (18.5%)	27 (29.0%)	<0.001
Number of stents	0.25 ± 0.72	0.60 ± 1.19	1.11 ± 1.42	<0.001
Gensini Score	0.00 (0.00–5.00)	6.00 (0.00–31.50)	24.00 (0.00–43.00)	<0.001
TG/HDL-C	2.05 (1.60–2.53)	2.64 (2.09–3.16)	3.23 (2.44–3.75)	<0.001

Groups were stratified by the tertiles of the TG/HDL-C ratio (T1 ≤ 1.11; T2 ≤ 2.02; T3>2.02). Continuous variables with a normal distribution were reported as mean ± standard deviation (SD), whereas skewed continuous variables were presented as medians (P25-P75). Categorical data are presented as n (%). BMI, body mass index; HTN, hypertension; DM, diabetes mellitus; SBP, systolic blood pressure; DBP, diastolic blood pressure; ALT, alanine aminotransferase; AST, aspartate aminotransferase; Scr, serum creatinine; TC, total cholesterol; TG, triglycerides; HDL-C, high-density lipoprotein cholesterol; LDL-C, low-density lipoprotein cholesterol; FBG, fasting blood glucose; HR, heart rate; cTnI, Cardiac troponin I; CHD, coronary heart disease; AMI, acute myocardial infarction; ACS, acute coronary syndrome; TG/HDL-C, the triglyceride to high-density lipoprotein cholesterol rate. The results demonstrated Gender, BMI, DM, SBP, ALT, AST, Scr, TC, TG, HDL-C, FGB, cTnI, CHD, AMI, ACS, Number of stents, Gensini Score and TG/HDL-C are different between the three groups (P<0.05).

### 5.5 Univariate and multivariate logistic analyses for predicting CHD in patients with NAFLD

The results of the logistic regression analyses, comparing T2 and T3 to the control group (T1), are presented in **Table 5**. In the unadjusted model, the risk of CHD was 1.29-fold higher in the T2 group (OR, 2.29; 95% CI, 1.26–4.16; P=0.006) and 3.0-fold higher in the T3 group (OR, 4.00; 95% CI, 2.18–7.37; P<0.001) compared to the control group.

**Table 5:** Univariate and multivariate logistic regression analyses of tertiles CHD risk for the TG/HDL-C ratio.

	Non−adjusted		Model I		Model II	
	OR (95%CI)	P	OR (95%CI)	P	OR (95%CI)	P
T1	Ref.		Ref.		Ref.	
T2	2.29(1.26–4.16)	0.006	2.16(1.13-4.13)	0.020	2.17(1.07-4.38)	0.031
T3	4.00(2.18–7.37)	<0.001	3.73(1.92-7.26)	<0.001	2.84(1.36-5.94)	0.005

Non-adjusted, unadjusted model; Model I, Model 1 adjusted for BMI, age, sex, smoking, hypertension, and diabetes mellitus; Model II, Model 2 adjusted for BMI, age, sex, smoking, hypertension, AST, ALT, TC, diabetes mellitus, Scr, FBG, and HR.

After adjusting for BMI, age, sex, smoking, hypertension, diabetes mellitus, AST, ALT, TC, Scr, FBG, and HR, the TG/HDL-C ratio, as a categorical variable, remains an independent risk factor for CHD risk among NAFLD patients (P<0.05).

After adjusting for BMI, age, sex, smoking, HTN, and DM in Model 1, the risk of CHD increased by 2.16-fold in the T2 group (OR, 2.16; 95% CI, 1.13-4.13; P=0.020) and 3.73-fold in the T3 group (OR, 3.73; 95% CI, 1.92-7.26; P<0.001) compared to the control group.

In Model 2, after further adjusting for AST, ALT, TC, Scr, FBG, and HR, the risk of CHD increased by 2.17-fold in the T2 group (OR, 2.17; 95% CI, 1.07-4.38; P=0.031) and 2.84-fold in the T3 group (OR, 2.84; 95% CI, 1.36-5.94; P=0.005) compared to the control group.

These findings suggest that the risk of CHD in patients with NAFLD significantly increases with elevating TG/HDL-C ratios, independent of BMI, age, sex, smoking, HTN, AST, ALT, TC, DM, Scr, FBG, and HR.

### 5.6 Correlation of TG/HDL-C ratio and Gensini score in patients with NAFLD combined with CHD

#### 5.6.1 Linear correlation analysis of TG/HDL-C ratio and Gensini score

**Figure 2:**
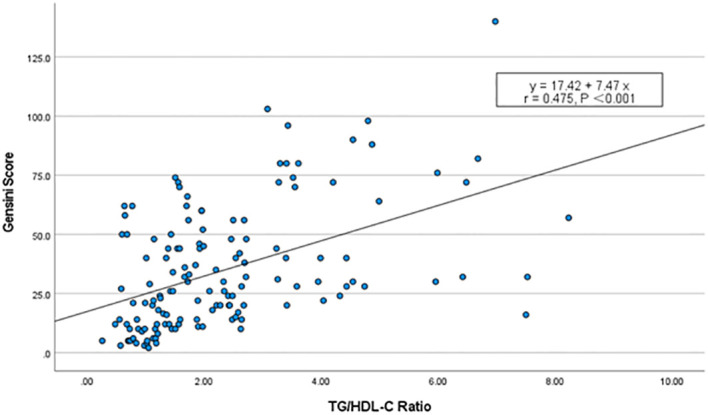
Scatterplot of TG/HDL-C ratio vs. Gensini score. Spearman’s correlation analysis found that there was a significant positive correlation between the TG/HDL-C ratio and the Gensini scores (r = 0.475, P < 0.001).

The severity of coronary arteries was assessed using the Gensini score based on coronary angiography results. Furthermore, we examined the potential linear relationship between the TG/HDL-C ratio and the Gensini score. Spearman correlation analysis revealed a significant positive correlation between the TG/HDL-C ratio and the Gensini score (r = 0.475, P < 0.001), suggesting a quantitative association. This finding further implies a linear relationship between the TG/HDL-C ratio and the severity of coronary arteries in patients with NAFLD (**Figure 2**).

#### 5.6.2 Univariate and multivariate linear regression analysis of TG/HDL-C ratio and Gensini score

After adjusting for various factors including age, sex, smoking, HTN, DM, Scr, FBG, and HR, the results indicated that a one-unit increase in the TG/HDL-C ratio was independently linked to a 7.75-unit increase in the Gensini score (β=7.75; 95% CI, 5.35–10.15; P < 0.001) (**Table 6**).

**Table 6:** Univariate and multivariate linear regression analysis of TG/HDL-C ratio and Gensini score.

Non−adjusted		adjusted		
	β (95%CI)	P	β (95%CI)	P
TG/HDL-C	7.47(5.13–9.81)	<0.001	7.75(5.35–10.15)	<0.001

Non-adjusted, unadjusted model; adjusted, adjusted for HTN, DM, Scr, FBG, and HR. After adjusted confounding factors, the TG/HDL-C ratio was independently linked to the Gensini score.

## 6 Discussion

NAFLD, a globally prevalent chronic hepatic metabolic disease, is strongly associated with an increased risk of coronary heart disease. Park et al. (13) prospectively studied the association between biopsy-confirmed NAFLD and atherosclerotic cardiovascular disease (ASCVD) over a 10-year period, revealing an independent correlation between NAFLD and heightened ASCVD risk. Toh et al. (14) performed a meta-analysis aimed at elucidating the association between NAFLD and the risk of CHD. Their findings revealed that the likelihood of developing CHD escalates in correlation with both NAFLD and its severity.

While the precise mechanism by which NAFLD elevates the risk of CHD is not fully understood, recent studies indicate a concurrent presence of NAFLD and ASCVD in patients with IR (15). Insulin resistance might play a mediating role in the development of CHD among patients with NAFLD. NAFLD initiates lipid metabolism disorders, oxidative stress, chronic inflammation, and additional associated pathological processes, ultimately leading to the development of IR (16). Simultaneously, the progression of IR is significantly associated with an increased risk of CHD (17, 18).

Although the gold standard for diagnosing IR is the high insulin-glucose clamp test, its cost and complexity have hindered clinical application. As a result, insulin resistance markers such as TG/HDL-C ratio, the triglyceride-glucose (TyG) index, and the lipid accumulation product (LAP) exhibit good clinical feasibility and utility (19, 20). Patients with NAFLD often present with dyslipidemia, primarily characterized by elevated TG levels and reduced HDL-C levels, while LDL-C levels may or may not be elevated (21). Thus, the TG/HDL-C ratio serves as a reliable indicator of insulin resistance and also reflects the abnormal lipid metabolism in patients with NAFLD.

Numerous studies have demonstrated a strong association between the TG/HDL-C ratio and an elevated risk of cardiovascular disease. In a prospective cohort study encompassing 9368 patients from four Chinese cohorts followed over 20 years, a positive correlation was observed between an elevated TG/HDL-C ratio and an increased risk of ASCVD (22). A separate study, comprising 403,335 participants from the UK Biobank, explored the association between the TG/HDL-C ratio and cardiovascular disease (CVD)risk, ultimately finding a positive correlation between the baseline TG/HDL-C ratio and an escalated CVD risk (7). Both studies consistently indicate a robust association between the TG/HDL-C ratio and CHD. Our findings suggest that in patients with NAFLD, there is a significant association between the TG/HDL-C ratio and an elevated risk of CHD (OR, 1.944; 95% CI, 1.502–2.515; P<0.001). After excluding the confounding effects of age, sex, smoking history, hypertension, DM, Scr, FBG, and HR, the results indicate that the TG/HDL-C ratio serves as an independent predictor of CHD in patients with NAFLD (OR, 1.791; 95% CI, 1.344–2.386; P < 0.001). The risk of CHD is significantly increased in patients with NAFLD compared to the general population, highlighting the critical clinical importance of investigating the correlation between the TG/HDL-C ratio and this high-risk group. Accordingly, this study generated a ROC curve using the presence or absence of CHD in NAFLD patients as the test variable. The results suggest that the TG/HDL-C ratio has some predictive value for CHD risk in NAFLD patients (AUC, 0.667; 95% CI, 0.615–0.740; P < 0.001). It exhibits high specificity but low sensitivity in predicting the outcome. This suggests that the TG/HDL-C ratio is particularly valuable in predicting positive outcomes for CHD risk in NAFLD patients. It is worth noting that, despite the lack of significant differences in AUC score, sensitivity, and specificity between the TG/HDL-C ratio and those parameters considered separately for TG or HDL-C. The TG/HDL-C ratio also serves as a reliable indicator of insulin resistance, reflecting the relationship between insulin resistance and the risk of coronary heart disease in NAFLD patients, while simultaneously highlighting the association between lipid abnormalities and the risk of coronary heart disease in NAFLD patients.

The TG/HDL-C ratio also holds a certain predictive value for assessing the severity of CHD in patients with NAFLD. Zhang et al. (23) retrospectively analyzed the TG/HDL-C ratio levels in patients with coronary artery disease (CAD) confirmed by coronary angiography. They found that the TG/HDL-C ratio was significantly higher in patients with multivessel CAD compared to those with single-vessel CAD. Similarly, Wu et al. (8) confirmed that the TG/HDL-C ratio can predict the severity of CAD in patients, as assessed by the Gensini score. In our study, Spearman correlation analysis revealed a significant positive correlation between the TG/HDL-C ratio and the Gensini score (r = 0.475, P < 0.001). This suggests a linear correlation between the TG/HDL-C ratio and the severity of coronary artery disease in NAFLD patients. And this linear relationship persists independently of various factors, including age, sex, smoking history, HTN, DM, Scr, FBG, and HR. These findings highlight the importance of managing not only traditional risk factors but also the CHD risk linked to the TG/HDL-C ratio in patients with NAFLD. Zhao et al. (24) investigated the relationship between the TyG index and both the risk of CHD and the severity of CAD in NAFLD patients. The results showed that the TyG index is associated with the risk of CHD and the severity of coronary artery disease in patients with NAFLD. However, due to its susceptibility to dietary influences, the feasibility of the TyG index is limited in certain conditions.

## 7 Conclusions

The TG/HDL-C ratio was positively correlated with CHD risk and reflected coronary atherosclerosis severity in NAFLD patients. TG/HDL-C ratio is an emerging index reflecting IR that differs from previous evaluation standards. This suggests that enhancing insulin sensitivity may potentially contribute to mitigating coronary artery disease and improving clinical outcomes. However, further validation through larger-scale studies is required.

## 8 Limitations

Firstly, this study is an observational, single-center investigation with a limited sample size, thus requiring cautious interpretation of its findings. Being a retrospective study, it inherently bears certain limitations. Future multicenter, large-scale prospective studies are warranted for validation purposes. Secondly, this study excluded patients with severe NAFLD, including those with liver fibrosis and cellular necrosis. This exclusion might have resulted in an underestimation of the predictive value of the TG/HDL-C ratio for coronary heart disease. Lastly, this retrospective study did not take into account the impact of statins on lipid indices. Additionally, in patients with ACS, the routine administration of loading doses of statins, as per the management guidelines of Chinese chest pain centers, influenced the study outcomes.

## Data availability statement

The raw data supporting the conclusions of this article will be made available by the authors, without undue reservation.

## Ethics statement

The studies involving humans were approved by the Medical Ethics Committee of Shenzhen Traditional Chinese Medicine Hospital and the fourth Clinical Medical College of Guangzhou University of Chinese Medicine. The studies were conducted in accordance with the local legislation and institutional requirements. The ethics committee/institutional review board waived the requirement of written informed consent for participation from the participants or the participants’ legal guardians/next of kin because This was a retrospective study in which the history data were generated from the patients’ previous clinical consultations, and the study was conducted only by searching the medical record system to obtain the relevant history data.

## Author contributions

BC: Visualization, Software, Methodology, Investigation, Formal analysis, Data curation, Conceptualization, Writing – review & editing, Writing – original draft. YY: Writing – review & editing, Validation, Supervision, Software, Methodology, Investigation. MC: Writing – review & editing, Validation, Supervision, Software, Methodology, Investigation. YW: Writing – review & editing, Validation, Supervision, Software, Methodology, Investigation. XS: Writing – review & editing, Validation, Supervision, Software, Methodology, Investigation. PC: Writing – review & editing, Validation, Supervision, Software, Methodology, Investigation. XL: Writing – review & editing, Validation, Supervision, Software, Methodology, Investigation. YG: Writing – review & editing, Validation, Supervision, Software, Methodology, Investigation. TL: Writing – review & editing, Validation, Supervision, Software, Methodology, Investigation. CX: Supervision, Software, Methodology, Investigation, Writing – review & editing, Validation. BL: Writing – review & editing, Supervision, Software, Project administration, Funding acquisition, Conceptualization. QL: Writing – review & editing, Supervision, Software, Project administration, Funding acquisition, Conceptualization.
